# Towards a hybrid approach to unveil the Chimaira of neurosciences: philosophy, aperiodic activity and the neural correlates of consciousness

**DOI:** 10.3389/fnhum.2023.1245868

**Published:** 2023-10-13

**Authors:** Aristea I. Ladas, Triantafyllos Gravalas, Tom Stoneham, Christos A. Frantzidis

**Affiliations:** ^1^Department of Psychology, CITY College, University of York Europe Campus, Thessaloniki, Greece; ^2^Department of Philosophy, University of York, York, United Kingdom; ^3^School of Computer Science, University of Lincoln, Lincoln, United Kingdom; ^4^Laboratory of Medical Physics and Digital Innovation, Biomedical Engineering and Aerospace Neuroscience (BEAN), Faculty of Health Sciences, School of Medicine, Aristotle University of Thessaloniki, Thessaloniki, Greece

**Keywords:** consciousness, neural correlates, relational, aperiodic activity, philosophy

## Abstract

Contemporary theories of consciousness, although very efficient in postulating testable hypotheses, seem to either neglect its relational aspect or to have a profound difficulty in operationalizing this aspect in a measurable manner. We further argue that the analysis of periodic brain activity is inadequate to reveal consciousness’s subjective facet. This creates an important epistemic gap in the quest for the neural correlates of consciousness. We suggest a possible solution to bridge this gap, by analysing aperiodic brain activity. We further argue for the imperative need to inform neuroscientific theories of consciousness with relevant philosophical endeavours, in an effort to define, and therefore operationalise, consciousness thoroughly.

## 1. Introduction

Our lived world is what we consciously perceive it to be. Therefore, according to one of the most established sleep neuroscientists, without consciousness there is nothing (Tononi, 2004). At least, there is nothing for the person who lacks conscious perception of the external world and this is evident in brain damaged patients such as split-brain, blindsight and amnestic individuals. The study of such conditions early in the 1960s and 1970s paved the way for the scientific examination of visual consciousness, although consciousness as related to brain activity had attracted scientific interest long before that (LeDoux et al., 2020).

The most frequently used method to investigate how consciousness is reflected on a neural level is the electroencephalogram (EEG). This line of research suggests that oscillatory activity is one of the major prerequisites of consciousness (Ruhnau et al., 2014). Among the EEG oscillations, the alpha band is often regarded as the one of major importance, especially at the pre-stimulus window. However, a local view of the neural correlates of consciousness (NCC) is not regarded as an adequate one. Consciousness is hypothesised to emerge from the integration of neural activity from different brain regions which receive stimuli-related information (John, 2002). The study of functional connectivity interactions among resting-state brain networks (e.g., somatosensory and the default mode network or the integration of information flow among fronto-parietal regions) increased our understanding of the conscious perception of upcoming stimuli. Specifically, functional connectivity analysis of whole-brain networks during deep sleep and especially Rapid Eye Movement (REM) are widely studied as experimental paradigms of conscious awareness (Tagliazucchi and van Someren, 2017). Moreover, Valencia and Froese (2020) describe how the synchronisation of EEG oscillations is what brings about the informational integration and therefore sense of flow that characterise conscious experience. After all, the idea that synchronisation of neural activity is basic to the phenomenon of consciousness has long been supported by Crick and Koch (1990).

The integration of neural information, attributed to the cortical and thalamocortical circuits, is considered to constitute the top-down content of consciousness, whereas the neural circuits that control the arousal states, such as the diencephalon and the brainstem, are considered to modulate consciousness’ bottom-up mechanism; the crucial role of both these systems has been well established by studies on the effects of anaesthetic-induced unconsciousness and a synthesis of bottom-up and top-down neural pathways has been recently suggested to better explain the seemingly diverse effects of anaesthesia on conscious states (Mashour and Hudetz, 2017).

Numerous consciousness theories have been developed. In the field of neuroscience currently, the Information Integration Theory (IIT; Tononi, 2004) and the Global Neuronal Workspace Hypothesis (GNWH; Dehaene et al., 1998, 2003; Dehaene and Changeux, 2005) are the most prominent ones (for a review see Del Pin et al., 2021; Melloni et al., 2023). The prevalence of the IIT and the GNWH mainly lies in their effectiveness in producing testable hypotheses in the search of the NCC (Del Pin et al., 2021).

A core difference between these theories is that the GNWH views consciousness from an objective, third-person perspective, while the IIT also tries to operationalize a first-person subjective perspective (for a review see Seth and Bayne, 2022), therefore approximating the Aristotelian view of conscious experience, given the reflexivity that characterises his investigations of cognition and the mind (Caston, 2002; Smit and Hacker, 2020). As valuable to the investigation of consciousness as these theoretical formulations may be, still they involve limitations in empirically evaluating both the objective (access or representational) and the subjective (i.e., phenomenal) aspects of conscious operations. They also neglect the third quality of consciousness, long described by Aristotle and discussed by Stoneham (2008) in his Purely Relational account of perception: the relationship between the observer and the perceived object. In this paper, we will summarise these theories. We will then argue on the added value of investigating the brain’s aperiodic activity and we will suggest a hybrid approach to thoroughly investigate consciousness.

## 2. Current consciousness neurocognitive models and limitations

### 2.1. Global neuronal workspace hypothesis (GNWH)

The GNWH (Dehaene et al., 1998, 2003; Dehaene and Changeux, 2005) was developed from the Global Workspace cognitive theory (Baars, 1998), according to which consciousness of perceptual contents arises only when these are communicated to a widespread network of many local processors in the brain; it is this wide informational broadcasting that constitutes conscious experience (for a review, see Mashour et al., 2020). The experiential integration of past, present and future, characteristic of conscious experience, is achieved because the local processors involved in this global workspace include ones of memory (past), of attention and perceptual input (present) and of motor plans and evaluative systems (future). Any piece of information within this network can become conscious once it is selected, amplified and sent to the rest of the processors involved. The mechanism of such informational selection, amplification and transmission between different cortical sites is described by the GNWH, which added another network responsible for the aforementioned connectivity between the different cortical areas. This neuronal network, characterised by its wide distribution, is able to both receive bottom-up and to transmit top-down information to all areas involved, therefore selecting, amplifying and transmitting the content of the local processors in a non-linear manner. The activation, or ignition as it is called, of the GNW can be spontaneous during resting state or due to perceiving an external stimulus or even due to processing/executing a cognitive task. One of the main brain areas involved in this workspace is believed to be the prefrontal cortex (PFC), as well as other nearby areas, all of which are characterised by strong, high density bidirectional interconnectivity (Mashour et al., 2020).

Despite its empirically supported utility in locating possible NCC, the GNWH seems limited in addressing the Aristotelian perspective (On the Soul 3.2) though, in that the representational content of consciousness, as reflected in the NCC in this case, still cannot reflect the totality of its phenomenal quality (Caston, 2002).

More specifically Aristotle holds that consciousness is an intrinsic capacity of humans, hence an integral part of the body; therefore, we must be able to measure it (Smit and Hacker, 2020). However, consciousness is also a higher-order capacity (Caston, 2002). This suggests that it emerges from lower-level capacities and constituents, such as mental representations and cognitive functions like attention, perception, memory. It is well established that human cognition is hierarchically structured (Carroll, 1997), starting from lower-level elements and progressively increasing complexity to result in higher-order functions (Botvinick, 2008). Yet, regarding consciousness, the end result is much more complex and sophisticated than any other higher-order process, as it is unique in also involving a subjective or phenomenal aspect (Marchetti, 2022). Therefore, measuring only its composites, such as the NCC, cannot reveal consciousness on its totality.

### 2.2. Information integration theory (IIT)

Another model that emerged in an effort to embrace both the quantity and the quality of consciousness is IIT (Tononi, 2004, 2008). According to the IIT, the (a) high integration of (b) rich information constitute the basic phenomenological characteristics of conscious experiences (for a review see Tagliazucchi et al., 2013), termed quality and quantity of consciousness, respectively. Richness of information is related to the repertoire of possible states that a system can be in, as well as the ability to transition between those states, while integration concerns how the different parts of a system interact and influence each other’s states. The IIT postulates that consciousness is linked to the properties of a system’s intrinsic causal power to influence itself from the integration of rich information within the brain; it is the ability for integration at any given moment that defines the level of conscious, or nonconscious, experience. The causal relationships among the elements of the system shape the way information is integrated and give rise to the subjective qualities of consciousness. Therefore, phenomenal consciousness arises from the specific patterns of integrated information within a system. However, this integration, which is defined by φ, is not equal to the pieces of information that it involves, but above and beyond them (Tononi, 2004). Certain criticisms of the IIT have been put forward, with the most persistent ones being its alleged inexactness (i.e., inability to map a specific experience to its neural substrates), panpsychism (i.e., that any organism can potentially be conscious) and conflation of φ with consciousness (Gruber, 2022). Koch, one of the developers of more recent IIT versions, supports that the issue of inexactness is not an issue of the theory per sé but a methodological one. He also asserts that IIT’s panpsychism is true and needs to be viewed as a strength in that indeed many organisms may be conscious of themselves although our past theories could not explain this. After all, comparative research across species, as collectively described in Irwin et al. (2022), empirically supports the existence of consciousness in various non-human organisms. As for the issue of conflation of φ with consciousness, Koch denies this by arguing on how the IIT describes consciousness to be a causal power that is reflected in its identical causal structure unfolding from neural substrates. According to Koch, the best critique of the IIT is that it is almost impossible to calculate φ, at least for the highly complex human brain, as all the subnetworks of a network must be first evaluated (Gruber, 2022; for a review see Seth et al., 2006).

### 2.3. Comparison of the two theories

Although the GNWH and the IIT efficiently provide testable predictions, the fundamental assumptions of these perspectives regarding consciousness and its neural substrates are different (for a review see Seth and Bayne, 2022; Melloni et al., 2023). For example, according to the IIT, a conscious experience will be reflected in sustained neuronal activation throughout the duration of that experience; whereas the GNWH suggests an initial “ignition” or activation upon the entrance of related information into the workspace, followed by a decay. In addition, a fronto-parietal network combined with high sensory cortices is supported by the GNWH, whereas the “posterior hot zone” (i.e., parieto-temporo-occipital) is suggested to be the locus of conscious emergence by the IIT (for a review see Koch et al., 2016). Summarising, the GNWH taps on the physical substrates of consciousness, whereas the IIT also tries to embrace the Aristotelian perspective by adding phenomenal consciousness. However, it is also clearly suggested that consciousness is above and beyond its NCC; yet our current methods do not allow us to measure or calculate (the φ in IIT) what could exist beyond the periodic activity of the neural substrates of consciousness. Or do they?

## 3. Aperiodic activity

The signals that are transmitted when functional connectivity occurs are distinguished in two types of electrophysiological activity, periodic and aperiodic. While periodic activity consists of fruitful waves of signals regarding the information that is passed from a neural hub to another, scale-free asynchronous or aperiodic activity (AA) is traditionally considered as noise between those waves. Therefore, for many years researchers have been applying filters and elegant methodological protocols in order to isolate AA from the recorded EEG signal, considering it as simple noise that carries no useful information (for a review see He, 2014). Nevertheless, we have reached a technological level that allows us to have a much sharper perspective of AA. Nowadays, we can safely support that AA is not just noise, but consists of many different frequencies used by the brain to functionally communicate, echoing simultaneously (He et al., 2010).

The main characteristic that delineates AA from simple noise is the dynamic nature of this non-linear activity, that is characterised by an 1/f-like slope regarding the frequency domain. Often named as “scale-free” activity, such temporal dynamics are seen in several natural phenomena such as earthquakes (Baiesi and Paczuski, 2004), forest fires (Nicoletti et al., 2023), biological networks (Almaas and Barabási, 2006) and many more. Such dynamics are believed to carry useful information regardless of the underlying power law principles (He et al., 2010; He, 2014), although the fact that periodic and aperiodic activity overlap makes it difficult to distinguish them (Donoghue and Watrous, 2022). The narrow-band oscillations that are responsible for the synergy of different brain areas are limited in transmitting long range signals. However, distant neuronal hubs firing simultaneously could dynamically produce AA (He et al., 2010), which enables the assessment of distant network cooperation such as the one described by the GNWH.

Considering that structural connectivity shapes, in part, functional connectivity (Honey et al., 2010; Babaeeghazvini et al., 2021), structural differences could also induce changes in the aperiodic components of one’s functional connectivity, hence in the subjective experience of consciousness. Regarding the structural advantage that AA has in terms of connectivity, wide-spread aperiodic perturbations, termed neuronal avalanches, have been recently suggested to convey individualised information that is highly specific to the person experiencing them, hence the alternative term “brain fingerprint” (Sorrentino et al., 2022). We therefore believe that the subjectivity inherent in consciously experiencing the world could be reflected physically in this AA.

### 3.1. Aperiodic activity to study consciousness

The informative value of AA is supported by Donoghue et al. (2020) and Donoghue and Watrous (2022) who argued for revealing AA’s parameters and analysing it separately and explicitly, given its physiological relevance and its clinical, cognitive and demographic correlates (Donoghue et al., 2020). The significance of AA’s contribution in the investigation of consciousness, is implied by the relations of variations in AA with different attentional states and stimulus properties (Waschke et al., 2021). That is, during different consciousness states, the state of attention also differs by being either externally directed (i.e., alert wakefulness) or internally (i.e., dreaming; for a review see Dixon et al., 2014); similarly, perceived stimulus properties, or better yet quality, also vary between dreaming and wakefulness. Given that AA reflects variance in both attentional states and stimulus properties then, we would expect that AA will also pinpoint these differences when analysed for wakefulness versus dreaming and therefore function as a proxy of differential states of consciousness.

#### 3.1.1. Previous studies on aperiodic activity in different consciousness states

**Figure 1:**
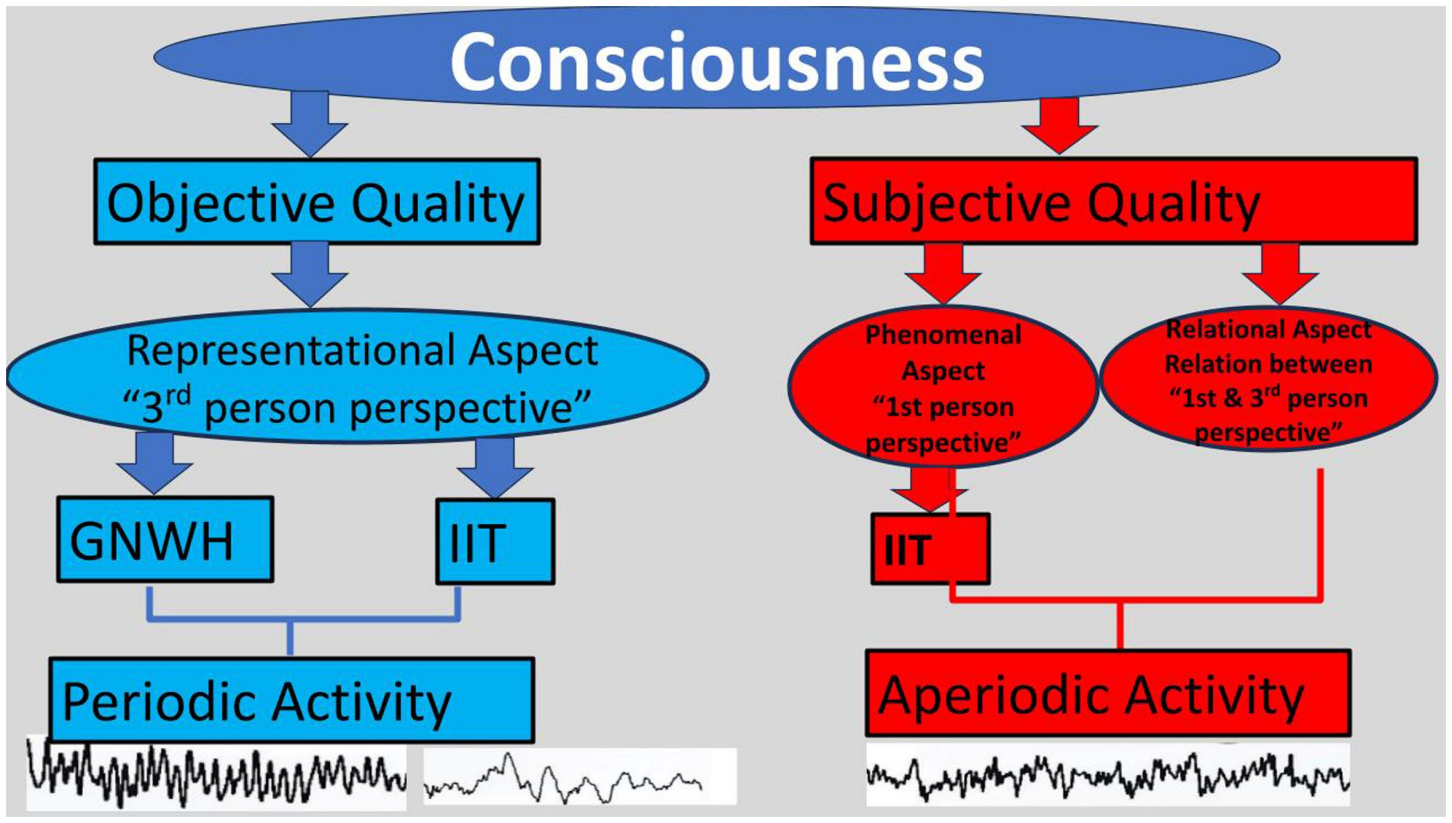
The combination of periodic and aperiodic activity to thoroughly investigate consciousness.

A series of recent studies have demonstrated the utility of AA as an electrophysiological marker of different consciousness states. That is, AA effectively differentiated between resting-state wakefulness, NREM sleep and anaesthesia (Muthukumaraswamy and Liley, 2018; Colombo et al., 2019; Miskovic et al., 2019; Lendner et al., 2020; Waschke et al., 2021; Zhou et al., 2021). Additionally Rabuffo et al. (2022) support that aperiodic or brain spontaneous activity (i.e., neuronal avalanches) reflects the concept of ignition postulated by the GNWH, by signalling the access of information into consciousness. They proceed in arguing for the combination of both oscillations and avalanches fora complete account of consciousness. The explicit analysis of AA could also potentially contribute to the IIT: Returning to its basic notion that φ is above and beyond its informational elements, it could be that AA represents this qualitative excess, since periodic activity explains it inadequately. Moreover, Kitchener and Hales (2022) argue that maybe consciousness does not emerge with sudden onsets, but could be there all along. Interestingly, it is the brain’s AA as opposed to oscillatory activity, that is, ongoing (e.g., van Heumen et al., 2021). This suggests that there could be valuable information hidden in the aperiodic component (see Figure 1).

## 4. Concluding remark: a hybrid perspective of consciousness

**Figure 2:**
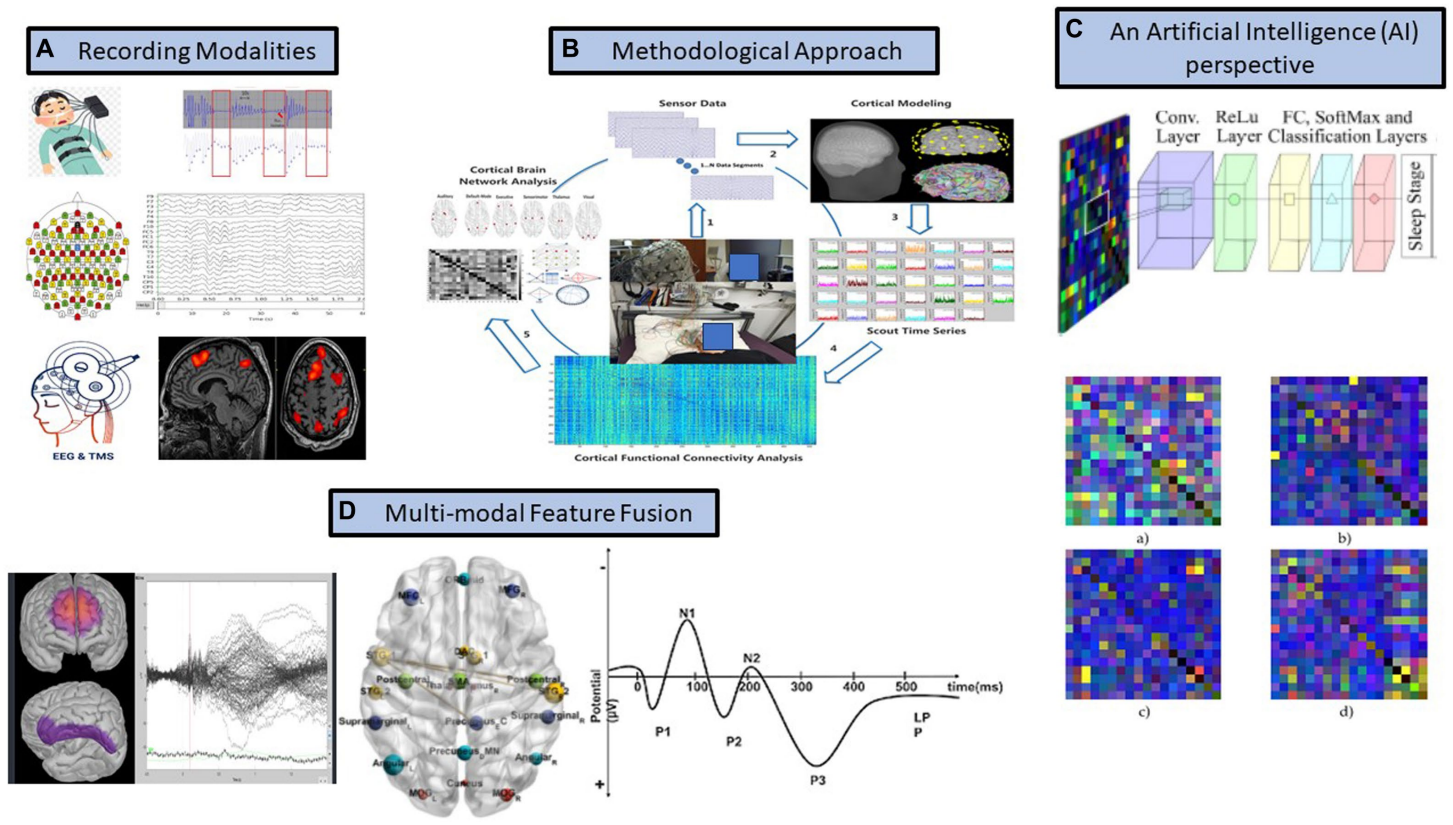
Our suggested methodology to assess consciousness. EEG = electroencephalography; TMS = Transcranial Magnetic Stimulation.

So far, we have presented arguments supporting the use of AA analysis to aid in thoroughly revealing the NCC, as periodic activity does not seem to explain it sufficiently. More specifically, we would like to shift the attention from traditional sleep research to the AA observed during the transition from slow wave activity to REM sleep and/or from drowsiness to the N1 sleep stage. Contemporary mathematical tools such as the Orthogonal Discrete Wavelet Transform (ODWT) offers a parameter free, excellent spatio-temporal analysis framework, which can identify the contribution of each EEG rhythm in very short time windows with high accuracy. When combined with functional connectivity analysis, it will also identify the dynamic interplay between different electrode sites (sensor level) or cortical regions (cortical level) (see Figure 2B). Further employing graph theory and network neuroscience may quantify the information flow and regions of high processing capacity, which will provide a hierarchical insight into the organisation of brain networks (modularity analysis and identification of important nodes / hubs) (see Figures 2C,D). This would be benefitted from employing high density electroencephalography (hD-EEG) ideally combined with either a neuroimaging modality (e.g., functional magnetic resonance imaging / fMRI) or a brain stimulation technique (e.g., navigated Transcranial Magnetic Stimulation; see Figure 2A), thus offering the opportunity to test consciousness theories through connectivity analysis.

Notably Aristotle, as a genuine mind biologist, believed that everything we experience must be reflected physically in the body (Smit and Hacker, 2020). This could suggest that multi-organ (e.g., brain-heart-muscle) interactions with brain activity (see Figure 2A), potentially further combined with sleep-related biomarkers’ analysis, could provide an even more integrative view of human consciousness. This is in line with the emerging scientific field of Network Physiology, aiming at understanding the interactions between different physiologic systems and how such synergies influence behaviour (e.g., Bartsch et al., 2015).

Yet, crucial to inferring consciousness from its mere biological, in this case neural, correlates, is firstly understanding it. Explaining conscious experience from a first-person perspective by observing it from a third-person perspective can only be achieved with the use of logic. Because no matter how rigorous the scientific method we use, the phenomenal character integral to the nature of (un)conscious experiences can best be described from a philosophical perspective. This is why we believe that if we are to thoroughly investigate consciousness, we need an interdisciplinary approach combining neuroscientific methods with philosophical endeavours. In such a way, not only will we provide empirical data that comprehensively reflect (un)conscious experiences, but we will also be able to ascribe meaning to those data, therefore understanding both the physical and the phenomenal properties of consciousness.

## Data availability statement

Publicly available datasets were analyzed in this study. This data can be found here: the original contributions presented in this perspective article are included in the references, further information can be directed to the corresponding author.

## Author contributions

AL, TG, TS, and CF contributed to the conception of the work. AL, TG, and CF contributed to the outline of the manuscript. AL wrote the first draft, led the literature review and the writing of the manuscript. TG contributed to the literature review and wrote sections of the manuscript. TS and CF contributed substantially in revising it critically for important intellectual content. All authors contributed to the article and approved the submitted version.
